# Supplementary material for: Antibiotic Resistance, Biofilm Formation, and Presence of Genes Encoding Virulence Factors in Strains Isolated from the Pharmaceutical Production Environment
Source: Pathogens. 2021 Jan 27;10(2):130. doi: 10.3390/pathogens10020130 (PMC7911615; doi:10.3390/pathogens10020130)
Supplement: Supplementary file 1 [file pathogens-10-00130-s001.pdf]

Supplementary 1

Table S1. The qualitative identification of genes taking part in the *Quorum Sensing* (QS), genes encoding virulence factors, and biofilm formation rate in *Pseudomonas aeruginosa* strains isolated from the pharmaceutical environment. Results for the particular isolated strains.

Strain	Presence (+)/Absence (-) of QS Genes				Presence (+)/Absence (-) of Virulence Factors Genes				
	IasI	*IasR*	*rhll*	*rhlR*	*lasB*	*aprE*	*lysyl*	*toxA*	Biofilm Formation§
**PAO 1**	+	+	+	+	+	+	+	+	Strong
**PA-1**	+	+	+	+	+	+	+	+	Strong
**PA-2**	+	+	+	+	+	-	-	+	Strong
**PA-3**	+	+	+	+	+	+	+	+	Strong
**PA-4**	+	+	+	+	-	-	-	+	Strong
**PA-5**	+	+	+	-	+	-	+	+	Strong
**PA-6**	+	+	+	+	+	-	-	+	Strong
**PA-7**	+	+	+	+	+	+	-	+	Poor
**PA-8**	-	-	+	-	+	-	-	+	Poor
**PA-9**	+	+	+	+	+	+	+	+	Strong
**PA-10**	+	+	+	+	+	+	+	+	Strong
**PA-11**	+	+	+	+	+	+	-	+	Strong
**PA-12**	+	+	+	+	+	-	-	+	Strong
**PA-13**	+	+	+	+	+	-	-	+	Strong
**PA-14**	+	+	+	+	+	-	-	+	Strong
**PA-15**	+	+	+	+	+	-	+	+	Srong
**PA-16**	-	-	-	-	-	-	-	-	Moderate
**PA-17**	-	-	+	+	+	+	-	+	Strong
**PA-18**	-	+	-	+	+	+	-	+	Moderate
**PA-19**	-	+	+	+	+	+	-	+	Strong
**PA-20**	+	+	+	+	+	-	+	+	Strong
**PA-21**	+	+	+	+	+	-	-	+	Strong
**PA-22**	+	+	+	+	+	+	-	+	Strong
**PA-23**	+	+	+	+	+	-	-	+	Strong
**PA-24**	+	+	-	+	+	+	-	+	Strong
**PA-25**	+	+	+	+	+	+	+	+	Strong
**PA-26**	+	+	-	-	+	-	-	+	Strong
**PA-27**	+	+	+	+	+	+	+	+	Strong
**PA-28**	+	+	+	+	+	-	-	+	Strong
**PA-29**	+	+	+	-	+	+	+	+	Strong
**PA-30**	+	+	+	+	+	+	+	+	Strong
**PA-31**	-	-	-	-	-	-	-	+	Poor
**PA-32**	+	+	-	-	+	-	+	+	Moderate
**PA-33**	+	+	+	+	+	+	-	+	Strong
**PA-34**	+	+	-	-	+	+	+	+	Strong
**PA-35**	-	-	-	-	+	-	-	+	Strong
**PA-36**	+	+	+	+	+	+	-	+	Strong
**PA-37**	+	+	+	+	+	-	+	+	Strong
**PA-38**	+	+	+	+	+	+	+	+	Strong
**PA-39**	+	+	+	+	+	-	-	+	Strong
**PA-40**	+	+	+	+	+	-	+	+	Strong

**PA-41**	+	+	+	+	+	+	-	+	Poor
**PA-42**	+	+	-	+	+	+	-	+	Strong
**PA-43**	+	+	+	+	+	+	+	+	Strong
**PA-44**	+	+	+	+	+	-	+	+	Strong
**PA-45**	+	+	+	+	+	-	-	+	Strong
**PA-46**	+	+	+	+	+	+	-	+	Strong

§ Non-biofilm producer: OD ≤ ODc; Poor-biofilm producer: ODc < OD ≤ 2× ODc; Moderate-biofilm producer: 2× ODc < OD ≤ 4× ODc; Strong-biofilm producer: OD > 4× ODc.

Table S2. The qualitative identification of genes taking part in the *Quorum Sensing* (QS), genes encoding virulence factors, and biofilm formation rate in *Pseudomonas aeruginosa* strains isolated from the pharmaceutical environment. Summation of the experimental observations concerning the presence of QS and virulence factors genes

	**Presence of Genes in *P. aeruginosa* Strains %**
**Quorum Sensing Genes**	
lasI	84.8
lasR	89.1
rhlI	80.4
rhlR	80.4
**Virulence Factors Genes**	
lasB	93.4
aprE	52.3
lysyl	39.1
toxA	97.8

Table S3. The qualitative identification of genes taking part in the *Quorum Sensing* (QS), genes encoding virulence factors, and biofilm formation rate in *Pseudomonas aeruginosa* strains isolated from the pharmaceutical environment. Summation of the experimental observations concerning the ability to form biofilm by tested strains.

**Biofilm Producers**	**Strains %**
Strong	84.8
Moderate	6.5
Poor	8.7

Table S4. PCR detection of tested genes. Oligonucleotide sequences for identification of genes taking part in *Quorum Sensing* and genes encoding virulence factors in *P. aeruginosa* strains.

	Sequence (5′-3′)	Amplicon Size (bp)	Annealing Temp. (°C)
	***Quorum Sensing* genes**		
lasI	F:GGCTGGGACGTTAGTGTCAT<br>R: CCAGCGTACAGTCGGAAAA	274	59.8
lasR	F: AAGGACAGCCAGGACTACGA<br>R: CCGAGCAGTTGCAGATAACC	490	54.6
rhlI	F: GTTCGACCATCCGCAAAC<br>R: GTCTCGCCCTTGACCTTCT	354	60.9
rhlR	F: CATGGCACCTATCCCAAGG<br>R: GGTCCATTGCAGGATCTCG	396	60.9
	**Virulence factors genes**		
Alkaline protesae (*aprE*)	F: GCAGAACAAGCACCCTACTAC<br>R: AACAGGGGCTTGAACAGGTA	156	60.40
Elastase *(lasB)*	F: CGAGAATGACAAAGTGGAACTG<br>R: CGTAGGTGTACTTGCCGATCTT	218	60.0
Endopeptidase IV (*lysyl*)	F: GTCACCTACGACGGGCATAC<br>R: CGCTGAAATCGGAGAAGTAGTC	196	62.2
Exotoxin A (*toxA*)	F: GGAGCGCAACTATCCCACT<br>R: TGGTAGCCGACGAACACATA	150	56.3

Supplementary 2. PCR reaction conditions.

Specificity of the starters was checked in BLAST Assembled Ref Genomes https://blast.ncbi.nlm.nih.gov/Blast.cgi programme. The following PCR reaction mixture was used: buffer with $MgCl_2$ (1x), dNTP Mix (0.2 mM each), primer pair (10 μM), thermostable Taq polymerase (1U), DNA (100–200 ng) isolated from the tested *P. aeruginosa* strains. As a negative control, the reaction mixture with water instead of bacterial DNA was used. The positive control was a DNA sample isolated from the strain of *P. aeruginosa* PAO1.

Parameters of amplification cycles: denaturation in 94 °C for 3 min, hybridisation of starters (Table S1A), for 1 min and extending starters in 72 °C for 1 min, for 30 cycles.

Presence of the amplified products of PCR reaction was evaluated using electrophoretic separation in 1.5% agarose gel against the weight molecular marker. The electrophoresis was carried out within 40 min under constant voltage of 130 V. Visualization of the product was carried out in UV light in transilluminator (Syngen).
